# Well‐Being and Academic Achievement: Differences Between Schools From 2002 to 2010 in the Helsinki Metropolitan Area

**DOI:** 10.1111/josh.12691

**Published:** 2018-10-09

**Authors:** Sakari Karvonen, Kari Tokola, Arja Rimpelä

**Affiliations:** ^1^ National Institute for Health and Welfare, Social Policy Research Unit, PO Box 30 FI‐00271 Helsinki Finland; ^2^ UKK Institute for Health Promotion Research Tampere Finland; ^3^ University of Tampere, School of Health Sciences and Tampere Centre for Childhood, Youth and Family Research, University of Tampere Tampere 33014 Finland; ^4^ School of Health Sciences and Tampere Centre for Childhood Youth and Family Research, University of Tampere Tampere 33014 Finland; ^5^ Department of Adolescent Psychiatry Tampere University Hospital Tampere Finland

**Keywords:** academic achievement and health, socioeconomic status, Finnish schools, Finnish School Health Promotion Survey

## Abstract

**BACKGROUND:**

We studied school‐level differences in academic achievement and well‐being from 2002 to 2010 in the Helsinki Metropolitan Area, as well as the connection between academic achievement, well‐being, and socioeconomic composition.

**METHODS:**

The School Health Promotion Study covered 109 schools and 78% of schoolchildren (N = 100,413; aged 14 to 16 years). Depression was measured with the modified Beck Depression Scale and academic achievement with the grade‐point average. Trajectory analysis identified groups of schools that were as heterogeneous over time as possible.

**RESULTS:**

Six school trajectories differing in academic achievement and 3 in well‐being were observed. Differences between the trajectories persisted over time. Academic achievement and well‐being were related: schools on a poor achievement trajectory were more often on a low‐well‐being trajectory. The poor socioeconomic composition of students was more common in low‐academic achievement and well‐being trajectories.

**CONCLUSIONS:**

Academic achievement and well‐being differed between schools and are closely related to each other and to the school's socioeconomic composition. Differences between the schools did not increase over time. Educational policies aimed at reducing differences should address both academic achievement and well‐being.

There is strong evidence from health research that low education is related to higher morbidity and mortality.^1^, ^2^ By adolescence, poor academic achievement is strongly related to health, to compromising behaviors and to poorer perceived health.^3^ The extent to which a school as an institution or the national educational system contributes to this is unclear. Education research has had little interest in the health and well‐being of students even though school‐level effects on academic achievement have been widely documented.^4^ In health research, interest in exploring the effects of the school, as an institution, on students' health and well‐being has been growing. These studies suggest that schools may vary not only in terms of academic achievement, but also in relation to students' mental and physical health and health‐related behaviors, such as smoking.^5^, ^6^ Education and health are not separated only in research but also at the policy level. Even established international monitoring systems covering students have focused either on health (eg, World Health Organization‐HBSC Study, ESPAD) or academic performance (such as PISA, TIMSS, and PIRLS). Consequently, none of these manage to report simultaneous development and variations in health and cognitive competencies between schools.

Finland is a Nordic welfare state that has been built on a meritocratic ideology of supporting equal opportunities to all. In the realm of schooling this has meant that all compulsory level schools follow a national curriculum, while school catchment areas have been geographically determined by the area of residence. There is no tracking according to different academic achievement levels before the age of 16, which is the upper age for compulsory education. Whereas differences between schools have been large in international studies,^7^ in Finland these have been small, even when compared with the neighboring countries of Sweden and Norway, which have relatively similar school systems to Finland.^4^ The success of Finland in the Organisation for Economic Co‐operation and Development's Programme for International Student Assessment (PISA) studies has been attributed to the low variability of academic outcomes between schools.^8^

Recently a concern has arisen over sociospatial segregation in more urbanized areas. In the Helsinki Metropolitan Area, the latest analyses have shown relatively well‐defined spatial patterns of socioeconomic segregation.^9^, ^10^ Residential areas differ significantly according to the proportion of low‐educated population and the population with an immigrant background. Differences in the socioeconomic background of the students are the key drivers of the variation in academic achievement between schools.^11^ Even though in Finland differences in academic achievement have been low,^4^ the concern over sociospatial segregation has led to worries that schools may be differentiating as well. Exploring this potentially diversifying trend over time is the primary focus of our study.

A free choice from among schools was implemented in the Finnish educational system in the early 1990s, partly relaxing schools from recruiting exclusively from their catchment areas. Nevertheless, most children even in urbanized areas choose their neighboring school, though students are increasingly selecting their schools based either on reputation or extra curriculum. To respond to this competition most schools now offer more teaching in rarer languages like Spanish or Russian, more sports or sciences, which also contribute to the social composition of their student population.

Given that students' health measured as behaviors and perceived health is strongly related to their academic achievement, we can expect differences between schools also in terms of indicators of health and well‐being. Earlier studies that have sought to identify so‐called effective schools, represented by clearly goal‐oriented pedagogy and a high level of feedback to the students,^12^ have found that students in these schools also exhibit less health compromising behaviors.^13^, ^14^ These findings support the idea that when examining differences between schools, the focus should be placed likewise on the health and well‐being of students.

The purpose of the study was to analyze differences between schools in academic achievement and the well‐being of the students and if these differences increased or decreased over time. The second purpose was to analyze if academic achievement and the well‐being of students were interrelated at the school level and whether the socioeconomic composition of the school was related to the academic achievement and well‐being of the students at the school level. The setting is the rapidly growing and urbanizing Helsinki Metropolitan Area in Finland, with the increasing sociospatial segregation of residential areas. Study data come from the national School Health Promotion Study from the years 2002 to 2010 which covers most schools with grades 8 and 9 (14‐ to 16‐year‐olds) in the Helsinki Metropolitan Area.

## METHODS

### Participants

The (Finnish) School Health Promotion Survey, conducted every second year, is a classroom survey for 8th and 9th graders (aged 14 to 16 years) designed to examine health, health behaviors, and school experiences of Finnish teenagers.^15^ It covers most schools in the country and 78% of schoolchildren. Participants completed the questionnaire anonymously during a school lesson under the supervision of a teacher, who does not interfere with the responses. Participants were informed in both oral and written form about the nature of the study as well as the voluntary nature of participation, and returning the survey was taken as consent. The questionnaire took 30 to 45 minutes to complete and was then placed in an envelope, sealed, and returned directly to the research center.

In the Helsinki Metropolitan Area, schools participated in the period 2002 to 2010. Over time an increasing number of schools participated, but only those schools that participated in each of the study years (N = 109) were included. The number of students by study year is as follows: 2002, N = 18,861; 2004, N = 20,492; 2006, N = 20,233; 2008 N = 21,173; and 2010, N = 19,654. Special schools and schools with less than 20 respondents in any of the study years were excluded.

### Instruments

The well‐being score was composed of a 12‐item version of the R‐BDI, a modification of the 13‐item Beck Depression Inventory Scale, the Finnish version of which has been previously validated.^16^ In the R‐BDI, an introductory question and a positive response option for each item were added. Thus, the R‐BDI constructs a dimensional continuum in which positive mood and depressive symptoms are the 2 end points of the continuum. Each item had 5 response options characterizing the mood of the students, ranging from bright to severe depression on a scale of 1 to 5. A sum score based on the items varied between 12 and 60, so that those with a more depressed mood had higher scores. According to the R‐BDI classification, scores higher than 39 characterize severe depression and those ranging from 32 to 39 show moderate depression. The well‐being score used here is a reversed depression score, which was calculated by subtracting the depression score from the maximum depression score, giving the range 0 to 48, where higher scores mean higher well‐being.

The academic achievement score was based on the Finnish grading system used for assessing students' learning; the range is from 4 (fail) to 10 (excellent). The grade point average (GPA) was asked with the question: “What was the mean of your school grades (including all subjects) in your last report card?” with structured responses ranging from below 6.5 (lowest) to 10 (highest score), with half‐point categories. The measurement echoed the Finnish grade system. In each category the middle point was used as the GPA; in other words, for the lowest category the GPA (4‐6.4) was 5.2. School‐based measures were aggregated from individual responses and calculated by interpolating percentage points from a cumulative probability distribution.

### Data Analysis

Clustering of schools into heterogeneous groups over time was carried out by applying trajectory analysis. Trajectory analysis is a group‐based semi‐parametric method for identifying distinctive groups of trajectories and modeling the development of trajectories over time.^17^ Statistically, the trajectory analysis is based on finite mixture models. The aim was to find groups of schools that are as heterogeneous over time as possible, according to the outcome measure that was used (well‐being, academic achievement). For each model in the trajectory analysis, 5 percentile points of the outcome measure were explained with a cubic curve of 5 time points. The percentiles selected according to the distribution of the outcome measure were the 5th, 25th, 50th, 75th, and 95th percentile. The final number of trajectories for each outcome measure was selected according to Bayesian information criteria (BIC). Rootograms of the posterior class probabilities were also examined so as to visualize the goodness‐of‐fit. A trajectory analysis was performed in R version 3.1.2 18 with the package stepFlexmix.^19^, ^20^, ^21^

## RESULTS

### Differences Between Schools

**Figure 1:**
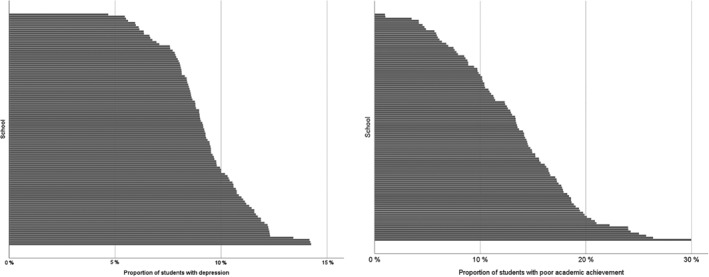
Percentage of Students Reporting Depression (Left) and Having Poor Academic Achievement (Right) in the Helsinki Metropolitan Area Schools, 2002 to 2010 Note. Each horizontal bar represents 1 school. Schools are presented from the lowest to the highest rate in both pictures.

Figure 1 demonstrates the rate of severe or moderate depression and the proportion of children with poor academic achievement (GPA < 7) in the schools for the period 2002 to 2010. The variation in both variables was considerable and ranged from 4.7% to 14.2% in depression and from 1.0% to 30.0% in poor academic achievement. The rates for each school were averaged over time to reduce random variation.

**Figure 2:**
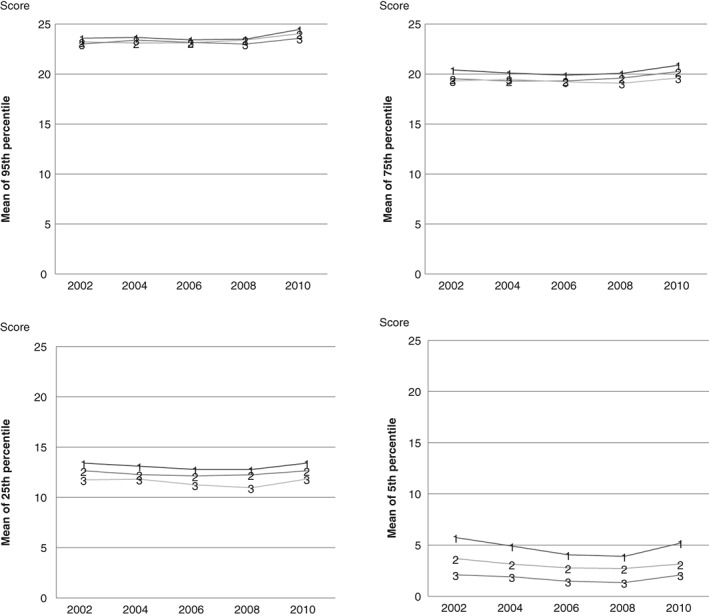
Means of Well‐Being Score of 3 School Trajectories (1, 2, 3) for 2002 to 2010

In the trajectory analysis, the schools clustered into 3 well‐being trajectories numbered 1, 2, and 3 in Figure 2. Figure 2 presents the differences between the trajectories in their well‐being score, which measures the mean of the school's percentiles in each trajectory. Means are calculated for 5th, 25th, 75th, and 95th percentile points of the well‐being score. The differences between the trajectories were negligible at the highest level of well‐being (95th percentile point of the score shown on the top left corner of the figure). The differences increased when moving toward low well‐being. At the low end, the differences between trajectories were clearly visible showing the difference of several score points between Trajectory 1 and Trajectory 3 (5th percentile point, bottom right corner). The differences between the trajectories remained rather stable over the study period.

**Figure 3:**
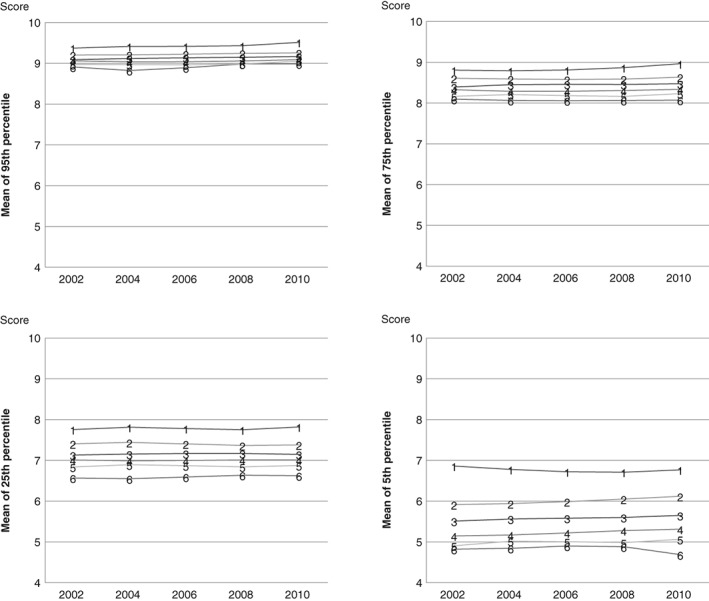
Means of Academic Achievement Score (GPA) of 6 School Trajectories (1 to 6) for 2002 to 2010

For academic achievement, the trajectory analysis produced 6 trajectories that are shown in Figure 3. Over time there were clear and distinct patterns between the trajectories in the academic achievement score, with practically no overlap between them. As with well‐being, the trajectories differentiated mostly in the poorest end of the distribution, the 5th percentile point. The differences were small in the higher end of the distribution (95th percentile point) but increased when the academic achievement became poorer. Changes in the differences between the trajectories over time were negligible.

### Differentiation of School Trajectories by Socioeconomic Composition

The socioeconomic composition of the schools differed consistently between the well‐being trajectories as well as between the academic achievement trajectories (Table 1). On average, there were more students with a disadvantaged socioeconomic background in the schools that belonged to poor well‐being or low‐academic achievement trajectories. The poorer or lower the trajectory, the higher the percentage of parents' unemployment, low level of education, and non‐nuclear families in the schools of the trajectory. Especially parents' level of education showed a strong association with academic achievement trajectories.

**Table 1:** Sociodemographic Composition of the Schools in Each Well‐Being Trajectory and in Each Trajectory of Academic Achievement (Percentage, Range, and Mean of Schools in the Trajectory)

Trajectory (Number of Schools)	At Least One of the Parents Unemployed	Neither of the Parents Have Higher Education	Parents Do Not Live Together
well‐being			
1 Best (24)	17.8% (12.5‐23.1)	40.6% (11.1‐73.3)	18.7% (10.1‐28.5)
2 (54)	21.0% (15.4‐27.7)	52.2% (27.6‐73.4)	23.7% (15.2‐31.7)
3 Poorest (31)	25.3% (16.0‐37.3)	54.8% (13.7‐77.5)	29.6% (16.6‐39.6)
Academic achievement			
1 Best (8)	18.2% (14.9‐22.8)	23.6% (11.1‐39.3)	19.9% (12.2‐28.5)
2 (20)	20.4% (13.8‐32.3)	35.8% (18.0‐50.9)	21.3% (15.2‐31.3)
3 (21)	19.1% (12.5‐24.1)	50.3% (28.8‐69.3)	21.8% (10.1‐35.7)
4 (31)	22.3% (14.8‐31.1)	55.1% (37.9‐73.4)	25.3% (15.9‐38.8)
5 (20)	22.4% (15.0‐27.9)	62.6% (52.4‐71.8)	25.8% (13.9‐34.8)
6 Poorest (9)	28.1% (22.4‐37.3)	63.5% (54.1‐77.5)	33.4% (25.6‐39.6)

### The Association of Well‐Being and Academic Achievement Trajectories

**Figure 4:**
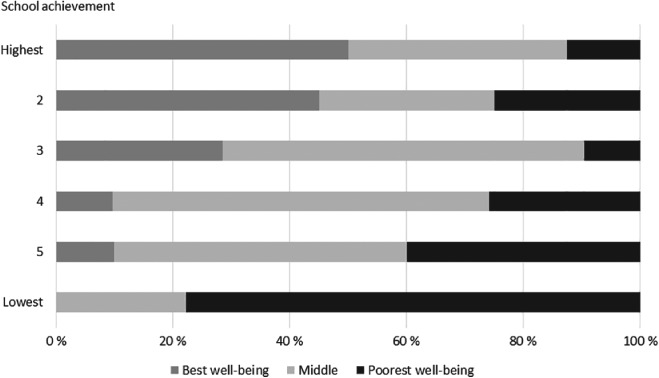
Percentage of Schools According to Well‐Being in Each Academic Achievement Trajectory (N = 109)

The association of well‐being and academic achievement trajectories is presented in Figure 4. The percentage of schools with poor level well‐being (black) increased when the level of academic achievement in the schools decreased, and vice versa, the percentage of schools with the best level of well‐being (dark gray) increased when the level of academic achievement increased.

## DISCUSSION

We studied differences in well‐being and academic achievement between the schools of the Helsinki Metropolitan Area in the years 2002 to 2010. The trajectory analysis showed 3 clusters of schools that differed according to their level of well‐being though the differences did not increase over time. For academic achievement, 6 clusters of schools differed on the level of the achievement but also here the differences remained the same over time. The socioeconomic composition of the schools with poor well‐being or low‐academic achievement comprised more disadvantaged students. Further, well‐being and academic achievement were closely related but not completely determined by each other at the school level.

There were no signs of increasing differences between schools in well‐being or academic achievement in the first decade of the 21st century. This means that the observed differences between schools have evolved earlier and maybe over a longer period of time. Why differences in academic achievement have not increased any more may reflect policy actions. The city of Helsinki, which is the biggest municipality of the area, has in particular adopted the policy of a needs‐based delivery of resources for schools. This has meant that resources have been redirected into those schools that face special challenges, such as a high level of deprivation or a high rate of immigrant students in the catchment area. Whether this has amounted to stable differences in well‐being cannot be answered by this study alone, but the strong association between well‐being and academic achievement in the schools suggests that the reasons could be similar.

We found well‐being and academic achievement to be associated quite strongly, which is exemplified by the fact that almost 80% of those schools performing the lowest academically belonged to the trajectory with the poorest well‐being. The association may partly reflect the association documented at the individual level: poorer performing students tend also to report poorer well‐being and vice versa. In the United States, eg, Ickovics et al^22^ report a strong cumulative association between health assets (such as body mass index or psychological well‐being) and academic achievement. However, this is hardly the whole story as there is a growing body of evidence showing differences between schools themselves that may contribute significantly to the student well‐being, thus explaining some of the association.^13^, ^23^, ^24^ Konu and Rimpelä^25^ identified school conditions (such as environment, organization, safety), social relationships (group dynamics, student‐teacher‐relationships etc) and means for self‐fulfillment (in other words guidance, encouragement) as contributing to school well‐being. Based on a similar reasoning, Markham et al^14^ reported greater substance use in “high value‐added” schools in Scotland, also showing that the school ethos contributes to health or health‐related behavior. Further, based on an extensive review of health problems affecting American urban minority youth Basch^26^ concluded that connectedness and engagement with school are key determinants of both academic achievement and adolescent health. We were not able to identify factors underlying the association, but further studies should explore more closely whether similar arrangements lie behind the 2 outcomes and whether they account for the association.

Our study showed that the socioeconomic patterning of well‐being and academic achievement between the schools were similar. The patterns reflect the area‐based social segregation of the families of the student population. In other words, a key finding of the study was that area‐based segregation was also associated with well‐being differences between schools and not only with academic achievement. Both the differences in well‐being and in academic achievement between the school trajectories were associated similarly with socioeconomic factors, such as parents' employment status, their level of education, and family structure. The indicator showing the strongest association with both outcomes was the level of the parents' education. This finding is in line with the review by Sellström and Bremberg,^5^ who concluded that pupils from schools where socioeconomic status (SES) is high on average perform better than pupils from low SES schools. An earlier multilevel study has also reported a small effect of high‐parental education on students' well‐being. 27

The earlier study suffered, however, from a same‐source bias that results from aggregating lower (individual) level data to a higher (school) level.^28^ We avoided this bias by limiting the study to the school level only, which, on the other hand, means that an ecological bias must be borne in mind. Some of the observed associations may reflect individual‐level correlations. Our focus was, however, not on assessing the “proper” level of effects but on identifying school trajectories and factors associated with the trajectories regardless of their etiology. In terms of assessing the outcomes of educational policies, it is more important to describe the trajectories between schools and to assess their stability than to disaggregate the outcomes by level. From this point of view, the fact that school trajectories were distinct and very stable points to a need to seek explanations for differences from among those factors that are independent of individuals, in other words the school level and beyond. In the United States, the recalcitrant nature of the problem has further been illustrated by pointing out that poverty is a third aspect in the reciprocal causal relationship between education and health.^26^, ^29^, ^30^ As each study year comprises different students, their individual characteristics cannot be the sole driver of the differences. Based on this reasoning, it appears that a school as an institution over and above the students' cognitive competence and family background does have effects on students' well‐being and academic achievement—the so‐called “value‐added” school effect. Similar “value‐added” effects may be possible to achieve in the United States, context as well by introducing a collaborative approach implemented at school level by a school health coordinator.^26^ Furthermore, an earlier review^31^ suggests that also in the United States improving health and increasing academic achievement of children and youth need to be viewed as a composite goal rather than separate goals that are responsibilities of different agencies.

The PISA studies have suggested that the particular strength of the Finnish educational system lies in the fact that it manages to cover all students well. This study supports the PISA assessments by showing that diversity between schools for example in academic achievements is not observed among students that perform well. In other words, even in those schools diverging from others on the basis of poor performance, well‐performing students achieve as well as those students from the schools that perform the best.^4^

### Limitations

Extending the analysis by considering the rate of immigrants would probably have thrown further light on the patterns found between the trajectories of schools, but this information was lacking from the data. The method of trajectory analysis allowed us to explore patterns of secular trends over time that would otherwise have been difficult to present or interpret. The trajectories may, however, hide deviant trends by single schools. To have a clearer view of the schools' potential contribution on these outcomes, it would be useful to explore these cases in more detail.

### Conclusions

The differences between the schools reflect the reputation or popularity of a school, the basis on which families select a school, and even their area of residence. This, in turn, results in favoring or aversion of some schools.^32^ Even though we could not show an increase in differences between schools over time, a widening area‐based social segregation in the future could also be expected to widen the differences between schools. To conclude, this study showed that well‐being and academic achievement are associated at the school level. Further, it showed that at the school level, family background was associated in broadly similar terms with differences in well‐being and academic achievement.

## IMPLICATIONS FOR SCHOOL HEALTH

Our study points out large differences between schools, not only in academic achievement of the students but also in students' well‐being. Academic achievement and well‐being in schools are closely related to each other and to the school's socioeconomic composition. Our study also points out that school differences mostly reflect the diversity at the poorer end of the well‐being and academic achievement distributions. This finding can be interpreted as meaning that to a student whose school performance is good or whose well‐being is high it matters very little which school he/she enters. Instead, it does matter for those who do not succeed well in academic tasks and whose well‐being is poor.

What kind of educational support and well‐being services schools offer, vary between countries and between schools. To mitigate accumulation of problems in academic achievement and well‐being, action should be taken on different levels.

- In the educational policy, the resources between the schools should be allocated not only according to indicators of academic achievement but also according to well‐being indicators. The close relationship between learning and well‐being shows that schools need both kinds of support services and these should work together.
- When school has a high proportion of students with poor academic achievement and a high proportion of students with poor well‐being, services for learning support, school health service and well‐being services should be integrated so that these services can tackle the problems together. School health coordinators who are cognizant of the different programs, services and policies along with their interlinkages could result a more cost‐efficient use of limited resources.
- In schools with lower level of students' well‐being, special efforts should be put on allocating resources to support students with more challenging backgrounds. School health services effectively promoting students' well‐being would most likely contribute to better educational outcomes.
- In all schools, coordination and exchange of information between teachers, health nurses, and doctors, as well as tutors should be promoted.

### Human Subjects Approval Statement

Informed consent was obtained from all individual participants included in the study. The study was approved (98,044, Minutes nr 2/98) by the ethics committee of Pirkanmaa Hospital District.
